# Physiological and molecular evidence of differential short-term heat tolerance in Mediterranean seagrasses

**DOI:** 10.1038/srep28615

**Published:** 2016-06-27

**Authors:** Lazaro Marín-Guirao, Juan M. Ruiz, Emanuela Dattolo, Rocio Garcia-Munoz, Gabriele Procaccini

**Affiliations:** 1 Integrative Marine Ecology, Stazione Zoologica Anton Dohrn, Villa Comunale, 80121 Napoli, Italy; 2 Seagrass Ecology Group, Oceanographic Center of Murcia, Spanish Institute of Oceanography C/Varadero, 30740 San Pedro del Pinatar, Murcia, Spain

## Abstract

The increase in extreme heat events associated to global warming threatens seagrass ecosystems, likely by affecting key plant physiological processes such as photosynthesis and respiration. Understanding species’ ability to acclimate to warming is crucial to better predict their future trends. Here, we study tolerance to warming in two key Mediterranean seagrasses, *Posidonia oceanica* and *Cymodocea nodosa*. Stress responses of shallow and deep plants were followed during and after short-term heat exposure in mesocosms by coupling photo-physiological measures with analysis of expression of photosynthesis and stress-related genes. Contrasting tolerance and capacity to heat acclimation were shown by shallow and deep *P. oceanica* ecotypes. While shallow plants acclimated through respiratory homeostasis and activation of photo-protective mechanisms, deep ones experienced photosynthetic injury and impaired carbon balance. This suggests that *P. oceanica* ecotypes are thermally adapted to local conditions and that Mediterranean warming will likely diversely affect deep and shallow meadow stands. On the other hand, contrasting mechanisms of heat-acclimation were adopted by the two species. *P. oceanica* regulates photosynthesis and respiration at the level of control plants while *C. nodosa* balances both processes at enhanced rates. These acclimation discrepancies are discussed in relation to inherent attributes of the two species.

Ongoing human-induced climate change are among the main threats affecting persistence and functioning of natural ecosystems^1^. Climate is predicted to experience not only a pronounced warming in the coming decades, but also a substantial increase in its inter-annual temperature variability, giving rise to more frequent, more intense and longer lasting summer heat waves^2^. These extreme thermal events intensify and prolong normal thermal stratification of marine waters and have been identified as the cause of massive mortality of sessile benthic key species^3^. Understanding how coastal key benthic species, in particular habitat-foundation species, respond to extreme heat events is imperative to predict how coastal ecosystems will respond to climate change^4^.

Sublittoral bottoms along the coasts of tropical, subtropical and temperate seas are dominated by seagrasses^5^, which are ecosystem engineers that structure one of the most valuable ecosystems in the biosphere, the underwater seagrass meadows^6^. Seagrass meadows produce goods and provide ecological services that are beneficial to humans and are key for the functioning of the marine coastal environment^7^. These valuable coastal ecosystems are potentially affected by anomalous heat events with critical consequences on their ecological and socio-economic functions and services^8^.

Increased mortalities have been reported for several seagrass species after recent summer heat waves^9^,^10^,^11^. Nevertheless, the experimental evidence of direct cause-effect relationship is not yet available, except for two *Zostera* species (see below), and little is known in general about the response of seagrasses to warming. It is assumed that seagrasses experience carbon imbalance under moderate heat stress due to a proportional higher increase in respiration than in photosynthesis, undergoing irreversible damage on their photosynthetic apparatus when the stress reaches critical levels^12^. Indeed, photosynthesis is the most heat sensitive key physiological process in higher plants^13^, involving various heat-sensitive components, as photosystems, the electron transport system, and CO_2_ reduction pathways. Heat-induced damage at any level may reduce the overall photosynthetic capacity of plants^14^. Beside this, the increased respiratory metabolism can potentially affect in the long term the plant carbon balance and the ability of plants for summer carbohydrate accumulation, on which plants depend for winter survival^15^. The effects of heat stress on seagrasses will, therefore, depend on the degree to which the photosynthetic and respiratory apparatus acclimate to adjust plant fitness and performance at the new growth temperatures^16^.

Although scarcely explored, the tolerance and resilience of seagrasses to heat stress produced during a summer heat wave is known to vary among species (*Zostera marina* and *Nanozostera noltii*)^17^ and among populations of the same species from contrasting latitudinal thermal environments (*Z. marina*)^18^. Inter- and intra-specific differences in heat tolerance of seagrasses is not surprising, considering that this group of plants comprises species with quite different ecological strategies and biological attributes^19^, and that individuals and populations of the same species are distributed, and likely thermally adapted, along ample clines (i.e. latitudinal and bathymetrical gradients). To this regard, comparative studies of the response of different species as well as of conspecific populations from the thermal extremes of the species distribution have shown very promising results for the identification of the underlying mechanisms of thermal stress tolerance in seagrasses^20^,^21^,^22^.

The Mediterranean Sea is particularly vulnerable to warming and the incidence of heat waves is expected to occur more frequently than in other regions^23^. Here the dominant seagrass species are the endemic *Posidonia oceanica* and the temperate *Cymodocea nodosa* that also grows in adjacent areas of the Atlantic Ocean. *C. nodosa* is a medium-sized and fast-growing seagrass that can be found in contrasting environments such as estuaries, coastal lagoons and open coasts, while the larger, long-lived and slow-growing species *P. oceanica* is only found in open coastal waters with more stable environmental conditions^24^. *C. nodosa* is therefore considered a pioneer seagrass with attributes characteristic of a eurobiotic species, while in contrast, *P. oceanica* behaves more like a stenobiotic organism. Accordingly, and in relation to warming, there is no evidence of negative effects on *C. nodosa* populations, whereas die-back was observed in several *P. oceanica* meadows following extreme hot summers^10^. Based on observed mortalities and water temperature projections, the functional extinction of *P. oceanica* meadows has been estimated to occur by the middle of the 21^st^ century^25^. The loss of the extensive and lush meadows formed by the species, which are considered the climax stage of the Mediterranean sublittoral environment, would have dramatic consequences for the whole Mediterranean ecosystem and coastal countries^26^. Meadows are distributed along a wide bathymetrical cline, with individuals within a single population living above and below the summer thermocline and thus experiencing distinct thermal regimens. This might have induced an adaptive differentiation through phenotypic plasticity and/or local adaptation, giving rise to shallow and deep ecotypes with distinct tolerance and acclimatization capacity to warming^27^. Indeed, the genetic differentiation evidenced between shallow and deep meadow stands (approximately above and below the thermocline), suggests a long-term selection by divergent environmental forces (e.g. temperature and light) of depth-adapted genotypes^28^,^29^. Consequently, *P. oceanica* plants from contrasting depths might manifest differences in heat tolerance and acclimatization capacities as was seen in other seagrass species but over larger geographical scales (i.e. across latitudes). The assessment of the response to warming of shallow and deep *P. oceanica* ecotypes is therefore a priority to improve our ability to predict their future trends in the framework of global climate change and to establish adequate management and conservation policies.

In the present study, we combined photo-physiological approaches with the analysis of the expression of genes encoding for the most thermo-labile components of the photosynthetic process, with the aim to assess short term heat tolerance and resilience of the two main Mediterranean seagrass species *P. oceanica* and *C. nodosa*. We also aimed to compare the differential response to heat stress among *P. oceanica* ecotypes living at the extremes of its depth distribution, to test whether ecotypes from contrasting depths differ in their acclimation capacity to heat stress. To this end, plants of both species and ecotypes were exposed to a short-term heat stress in a mesocosms system to study their response during and after stress through the assessment of expression of photosynthesis and stress-related genes, photosystem II (PSII) functionality, and photosynthetic and respiration performances.

## Results

**Figure 1:**
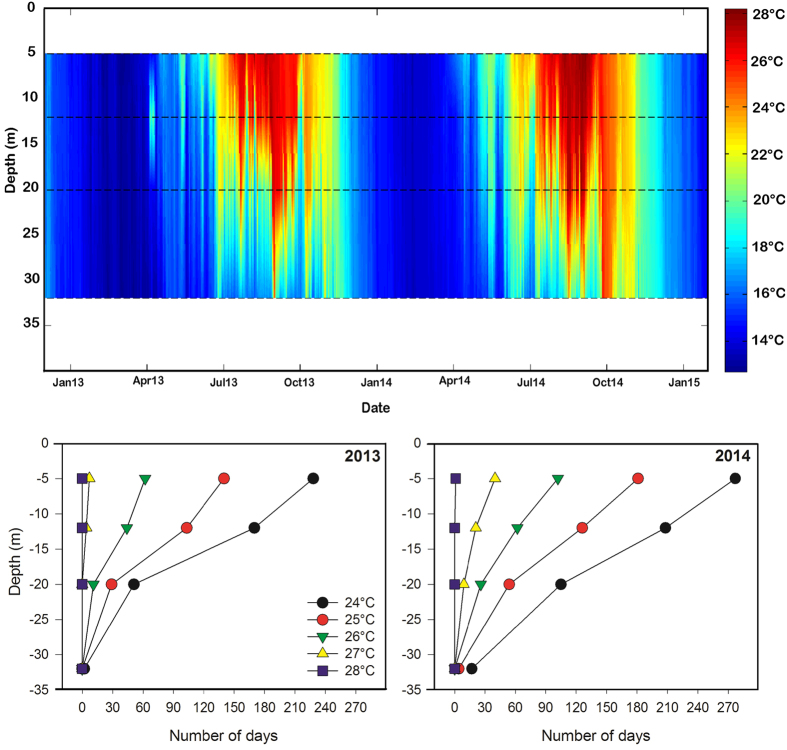
Water column temperature. Temperature registered along 2013 and 2014 in the sampled meadow (upper panel). Dashed lines represent the depth at which sensors were installed (i.e. 5, 12, 20 and 32 m). Number of days in 2013 and 2014 above a given temperature (i.e. from 24 to 28 °C) at the depths at which temperature sensors were installed (lower panels).

Water column above sampled meadows remained vertically mixed from mid-autumn to late spring when it begun to warm up generating stratification and fixing the thermocline (Fig. 1). Both the duration and the intensity of the thermal stratification varied between 2013 and 2014, being stronger and longer in the second year. Water temperature rapidly raised and kept warm during the summer period at 5 m depth, showing a more fluctuating pattern at 25 m depth. On an annual basis, the average (±SE) number of days that shallow plants (5 m) were exposed to temperature levels above 24, 25, 26 and 27 °C were 253 ± 25, 161 ± 21, 82 ± 20 and 23 ± 17, respectively. These plants experienced temperatures above 28 only during one day in 2014 (Fig. 1). In contrast, the estimated corresponding values for deep *P. oceanica* plants (25 m) were respectively 23, 19, 16 and 12% of shallow ones, and deep plants were never subjected to temperatures as high as 28 °C.

**Figure 2:**
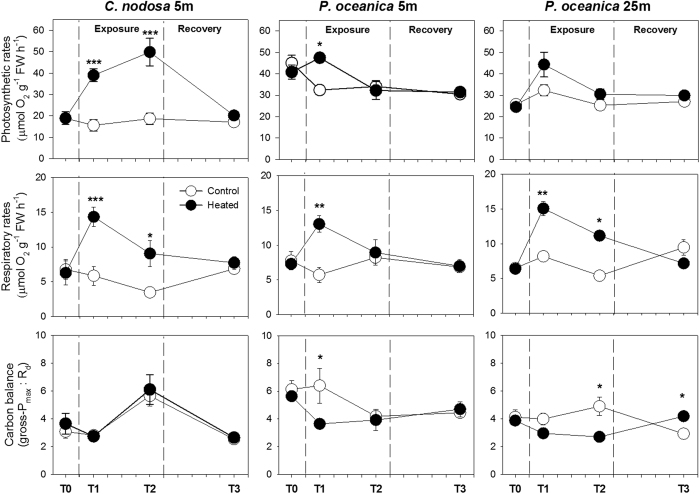
Photosynthesis Irradiance curve parameters. Photosynthetic rates (top), respiratory rates (middle) and leaf carbon balance (bottom) of shallow *C. nodosa* (left) and *P. oceanica* (centre) and deep *P. oceanica* (right) from the control (⚪) and heat stress (⚫) treatments along the course of the experiment. Bars represent SE± n = 4. Asterisks indicate significant treatment effects as identified in the *post-hoc* analysis. *p < 0.05; **p > 0.01; ***p < 0.001.

*C. nodosa* plants showed a significant increase in photosynthesis (2.5 and 2.6-fold in T1 and T2, respectively) and, in a similar proportion, in their respiratory activity, during the heat exposure (Fig. 2). In consequence, the leaf carbon balance of *C. nodosa* was unaffected during heat stress (Supplementary Table S1). Shallow *P. oceanica* plants also evidenced a significant increase in photosynthesis (1.5-fold), after one day of heat exposure, and a proportionally higher increase in respiration (2.3-fold), resulting in a 40% reduction of leaf carbon balance (Fig. 2; Supplementary Table S1). Nevertheless, leaf carbon balance recovered after 5d of heat stress, when photosynthesis and respiration equaled that of controls. Contrarily, heat-stressed deep *P. oceanica* ecotypes did not significantly increase their photosynthetic rate during the heat exposure, and since the 2-fold higher respiratory rates observed in T1 was not stabilized in T2, their leaf carbon balance progressively deviated from the controls (Fig. 2). The photosynthetic and respiratory activities of all heat-stressed plants returned to control levels after the recovery period.

**Figure 3:**
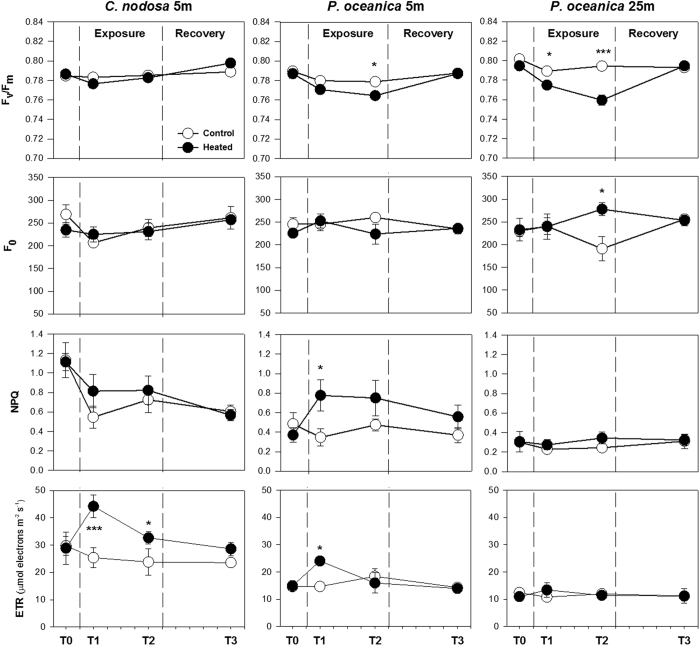
Chlorophyll *a* fluorescence parameters. Maximum photochemical efficiency of PSII (F_v_/F_m_; top), basal fluorescence (F_0_; second), thermal energy dissipation (NPQ; third) and electron transport rate (ETR; bottom) of shallow *C. nodosa* (left) and *P. oceanica* (centre) and deep *P. oceanica* (right) from the control (⚪) and heat stress (⚫) treatments along the course of the experiment. Bars represent SE± n = 4. Asterisks indicate significant treatment effects as identified in the post-hoc analysis. *p < 0.05; **p > 0.01; ***p < 0.001.

The imposed heat stress significantly affected the photochemical capacity of deep *P. oceanica* plants as evidenced by the significant and progressive reduction in F_v_/F_m_ (Fig. 3; Supplementary Table S1). In addition, these plants were the only ones that showed a significant increase in basal fluorescence (F_0_) after 5d of heat treatment (T2, Fig. 3). Shallow *P. oceanica* displayed effects on F_v_/F_m_ only in T2, when the reduction (2%) was similar to that observed in deep plants in T1. Contrarily, *C. nodosa* showed no heat effects on this parameter. Shallow *P. oceanica* were the only plants that increased thermal energy dissipation in PSII during the exposure to heat, being NPQ values significant only at the beginning of the stress (i.e. T1) when it doubled those of controls (Fig. 3).

Electron transport rates of heat-stressed plants showed a response pattern similar to photosynthesis. *C. nodosa* displayed a significant and marked ETR increase all along the exposure. For shallow *P. oceanica* plants, a significant ETR increase was present just in T1, while rates of deep plants were similar to controls (Fig. 3). Despite the observed heat stress effects on fluorescence parameters, they all returned to control levels after a recovery period of 5d.

**Figure 4:**
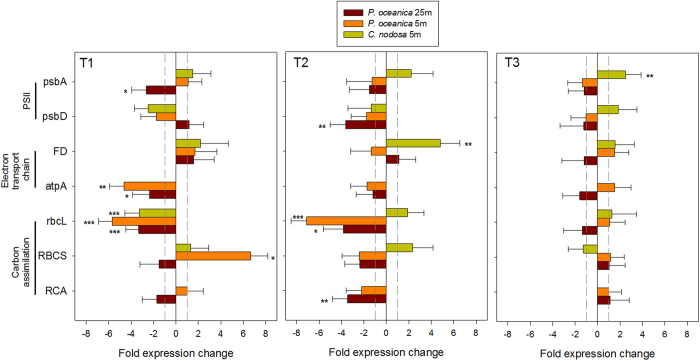
Level of expression of photosynthesis-related genes. Relative level of expression of genes selected in accordance with the three major sensitive sites in the photosynthetic apparatus (PSII: psbA and psbD; electron transport chain: FD and atpA; and carbon fixing processes: rbcL, RBCS and RCA) in shallow (5 m) *C. nodosa* and *P. oceanica* plants and deep (25 m) *P. oceanica* plants along the course of the experiment: T1 (24 h of heat exposure), T2 (5d of heat exposure) and T3 (5d of heat recovery). Error bars represent SE± n = 4. Significance levels are indicated: *p < 0.05; **p > 0.01; ***p < 0.001.

As concerns gene expression, the multivariate analysis (PCA) did not evidenced a common patter in the response to heat mainly due to constitutive interspecific differences that accounted for 87% of the total variance (Supplementary Figure S1). Heat-stressed *C. nodosa* plants showed little expression variation in selected photosynthesis-related genes and only rbcL (T1) and FD (T2) showed significant down- and up-regulation, respectively (Fig. 4). Responses were more acute in *P. oceanica*. While both deep and shallow plants significantly reduced the level of expression in rbcL, only in deep plants RCA was significantly down-regulated in T2, while in shallow plants RBCS evidenced 6-fold higher expression levels in T1 (Fig. 4, Supplementary Table S2). Both photosynthetic reaction center genes from PSII (psbA and psbD) showed significantly decreased mRNA levels only in deep *P. oceanica* plants.

**Figure 5:**
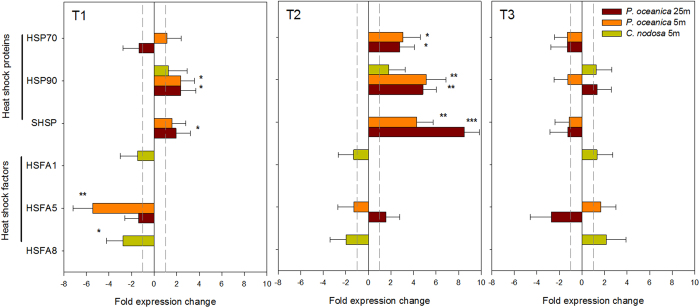
Level of expression of stress-related genes. Relative level of expression of heat shock proteins (HSP70, HSP90 and SHSP) and heat shock factors (HSFA1, HSFA5 and HSFA8) in shallow (5 m) *C. nodosa* and *P. oceanica* plants and deep (25 m) *P. oceanica* plants along the course of the experiment: T1 (24 h of heat exposure), T2 (5d of heat exposure) and T3 (5d of heat recovery). Error bars represent SE± n = 4. Significance levels are indicated: *p < 0.05; **p > 0.01; ***p < 0.001.

Regarding HSP genes, both *P. oceanica* ecotypes showed similar pattern of expression levels (Fig. 5). HSP90 significantly and progressively increased its level of expression from 2-fold in T1 to 5-fold in T2, when HSP70 was also similarly and significantly up-regulated (3-fold; Fig. 5; Supplementary Table S3). SHSP was also significantly over-expressed in T2, but with much higher levels in deep plants (8.5-fold) than in shallow ones (4.3-fold). Opposite pattern, however, was observed for the transcription factor HSFA5 that did not show significant expression level changes in deep ecotypes but was significantly down-regulated (5.4-fold) in shallow ones at the beginning of heat stress. In *C. nodosa*, neither HSP90 nor HSFA1 evidenced changes in their level of expression along the experiment and just HSFA8 was significantly down-regulated in T1.

## Discussion

### Intraspecific variability in P. oceanica heat tolerance

*P. oceanica* ecotypes coming from shallow and deep stands within the same meadow manifested different photo-physiological tolerance to the imposed heat stress, with deep plants evidencing a lower tolerance. Deep ecotypes displayed increasing stress-induced damage and adverse effects on the functionality of their photosynthetic apparatus during the heat exposure, as reflected by the progressive reduction in photochemical efficiency and the rise in basal chlorophyll *a* fluorescence (F_0_). The F_0_ increase, that is considered a very suitable test to assess the state of the photosynthetic machinery in terrestrial plants under heat stress^30^, confirmed that deep plants were experiencing critical temperature levels that lead to PSII inactivation^31^. PSII can suffer serious damage by the accumulation of reactive oxygen species (ROS) under thermal stressful conditions, which in turn can also inhibit the PSII repair cycle^32^. The suppression of genes encoding for the two PSII core proteins D1 (psbA) and D2 (psbD) points to the inhibition of the PSII repair cycle in deep *P. oceanica* plants, since *de novo* synthesis of D1 protein, in particular, is imperative for the restoration of damaged PSII^33^.

In contrast, PSII repair cycle of shallow heated plants was unaffected, as evidenced by unaltered psbA and psbD mRNA levels, preventing the appearance of heat-induced PSII damage. The higher PSII thermal stability of shallow plants seems to be related to their ability to activate, in the early stages of heat stress, the photoprotective mechanism associated to the xanthophyll cycle pigments (NPQ increase), which are also considered strong ROS scavengers^34^. PSII functionality was further protected from damage by the heat-stimulated photosynthetic electron flow (i.e. ETR increase) that alleviates excessive PSII excitation pressure under stressful conditions^35^. This response also drove photosynthesis enhancement to compensate the sharp heat-induced respiratory increase and partially reestablishing the leaf carbon balance at the beginning of the heat exposure^16^. The marked induction of one Rubisco small subunit (RBCS), coupled with the increasing (2 fold) of Rubisco activase (RCA), which is known to positive affect plant’s productivity under high temperatures^36^, could explain this photosynthetic enhancement. Indeed, deep *P. oceanica* ecotypes, contrary to shallow ones, experienced a significant suppression of those genes encoding key elements of the electron transport chain (atpA) and of the carbon fixing processes (rbcL, RBCS and RCA) being at the same time unable to enhance their electron transport and photosynthetic rates. These early molecular alterations, which are considered clear symptoms of photosynthetic heat sensitivity in terrestrial plants^37^, can be regarded as molecular evidences of the greater sensitivity of deep *P. oceanica* to heat stress.

Warming induced overexpression of HSP70 and HSP90 in plants from both depths in accordance with their role to re-establish normal protein conformation and thus cellular homeostasis^38^. Nevertheless, deep plants overexpressed SHSP at much higher levels than shallow ones, likely reflecting higher PSII damage and stress level since chloroplast-localized SHSP acts to protect PSII under stress conditions^39^. Moreover, only shallow plants evidenced a strong down-regulation in the level of expression of the transcriptional factor HSFA5 at the very early stages of stress. This response could be the molecular basis for the differential heat tolerance between both *P. oceanica* ecotypes, as heat shock transcriptional factors control the expression of many other genes for the acclimation of organisms to acute stresses^40^. In particular, HSFA5 acts as specific repressor of HSFA4, which is a potent activator of heat stress gene expression in terrestrial plants^41^. Therefore, its inactivation in shallow plants would allow the factor A4 to activate the molecular responses needed for a successful acclimation to the imposed stress. The potential usefulness of these transcriptional factors as early warning indicators of heat stress in the species seems promising and should be further investigated.

After several days under heat, shallow ecotypes evidenced thermal acclimation by reestablishing the balance between leaf respiration and photosynthesis, whereas the leaf carbon balance of deep plants progressively decreased due to their inability to stabilize respiration. Respiratory homeostasis is a functional trait associated with heat tolerance in terrestrial plants that allows to balance photosynthetic carbon gains and respiratory carbon losses^16^ and represents a key mechanism for attaining a successful heat acclimation in shallow *P. oceanica*. This acclimative capacity to short-term heat stress is consistent with the absence of clear detrimental effects on growth and survival of shallow *P. oceanica* plants after longer exposures^42^. On the other hand, in spite of the incapacity of deep *P. oceanica* to acclimate to heat and of the accumulated PSII heat-induced damage, plants were able to recover after a recovery period of several days evidencing that the injury experienced during the short-term heat exposure were reversible once the stress ceased. However, we cannot exclude that if stress lasts longer, heat induced-damage may be irreversible, causing plant death as observed at the lower limit of *P. oceanica* meadows after extreme hot summers^43^.

### Interspecific variability in heat tolerance between the two key Mediterranean seagrasses

*P. oceanica* and *C. nodosa* from the same thermal environment have displayed different degrees of heat stress on photosynthetic performance and different responses to attain successful heat acclimation. While acclimation in *P. oceanica* resulted, after several days of warming, in complete metabolic homeostasis (i.e. identical rates of photosynthesis and respiration between heated and control plants), acclimation in *C. nodosa* resulted in the balance between heat-enhanced photosynthetic and respiratory rates^44^. These contrasting heat acclimation responses reflected the interdependence between both processes, photosynthesis depending on compounds produced during respiration (e.g. ATP) and respiration relying on photosynthesis for substrate^45^, and can be explained on the basis of the different biological attributes of the two seagrass species. The higher carbohydrates content of *C. nodosa* leaves, in respect to *P. oceanica*^46^, which is considered an important physiological trait associated with heat stress tolerance^47^, would allow the species to maintain enhanced respiration over longer time periods, thanks to a higher availability of respiratory substrates.

*C. nodosa* also showed the ability to maintain elevated photosynthetic electron flow to sustain enhanced photosynthesis all along the heat exposure, which could be considered an advantage to grow and persist in more confined, shallow environments with high thermal stress (e.g. coastal lagoons)^48^. The increased expression in this species of ferredoxin (FD), which is a key component of the photosynthetic electron transport chain, can be interpreted as a response to favor a higher electron transport capacity increasing NADPH, which together with the ATP will drive photosynthesis enhancement^49^. *P. oceanica*, instead, is only capable to sustain enhanced photosynthesis in the very early stages of heat exposure, likely due to the small reduction observed on their photochemical capacity after several days of heat stress. In accordance, the level of expression of putative photosynthesis-related genes was barely altered by warming in *C. nodosa* whereas they were highly modified in *P. oceanica*. This differential photosynthetic heat response is consistent with the evolutionary history of the species, since the *Cymodocea* genus has a tropical distribution, with *C. nodosa* being the only temperate member, while all the species of the *Posidonia* genus live in temperate areas, both in Mediterranean and along the central-southern Australian coasts^5^.

Acclimation discrepancies between the two seagrasses can also be related with inherent biological attributes of fast- and slow-growing plant species as for instance the ratio above/below ground tissues and developmental plasticity^50^. Considering that non-photosynthetic below-ground tissues also experience heat-induced respiratory increase, increments in below-ground carbon demand will cause whole plant carbon imbalance in a greater extent in species with higher below-ground biomass as *P. oceanica*^51^. For this species, it would be more convenient to achieve complete respiratory homeostasis due to its very low photosynthetic:non-photosynthetic biomass ratio and to the tight dependence of the species from rhizome carbon reserves for overwintering and re-growing^52^. Contrarily, *C. nodosa* has much higher above:below biomass ratio and also a different carbohydrate energy storage strategy that favors species’ resilience even under long periods of light deprivation^53^. The strategy of balancing photosynthesis and respiration at increased rates is also achieved by modifications in the ratio between above- and below-ground biomass under longer heat exposures^42^ as allowed by the high morphological and growth plasticity of the species.

### Ecological implications of differential heat tolerance in Mediterranean seagrasses

In the studied meadow, depth and shallow *P. oceanica* ecotypes experience different thermal regimes due to the formation of the summer thermocline that is reestablished every year but with duration and intensity that can vary according to the prevailing atmospheric conditions. These seasonal thermoclines force shallow plants to experience higher temperatures and over longer time periods than deep ones, and play a critical role in determining the bathymetric distribution of sessile species in accordance with their thermal tolerance^54^. Although it is hard to conclude that adaptive evolution has locally occurred in *P. oceanica* and to disentangle it from phenotypic plasticity^27^, the recurrence and persistence of this thermal gradient may have imposed divergent selection on the species providing fitness advantage under these contrasting local conditions^55^. Shallow *P. oceanica* plants living in a thermal fluctuant environment better tolerate heat stress and are thus less affected by extreme heat events; while deep plants that evolved in relatively constant and colder environments are more sensitive. This suggests that *P. oceanica* ecotypes within the same population are thermally adapted to their local conditions^56^, which is not surprising given the clear genetic differentiation existing between shallow and deep meadow sections within continuous *P. oceanica* meadows^28^. Recent studies on gene expression have also suggested the adaptation of shallow and deep *P. oceanica* ecotypes to existing conditions (e.g. light and temperature) above and below the summer thermocline^29^. The adaptive population divergence may have been fostered by the low occurrence and success of sexual reproduction in the species and its asynchrony between both meadow stands^57^,^58^. Local adaptation, in fact, is promoted when the spatial scale of gene flow is small relative to the scale over which the selective gradient varies, being more likely to occur in clonal plants such as seagrasses^59^. The small-scale adaptation of the species would have important consequences for its conservation and management and would help to improve the accuracy of predictions about the impacts of climate change on these valuable ecosystems. If deep meadow stands are expected to regress as a consequence of the more frequent extreme thermal events, stress-resistant shallow genotypes could be used for assisted colonization or restoration of deep meadow margins. This, however, may have implications on the genetic diversity of natural populations and on the *P. oceanica* metapopulation as a whole with unknown implications on the ability for the species to cope with future environmental changes^9^.

On the other hand, *P. oceanica* and *C. nodosa* plants from the same shallow thermal regime both attained short-term heat acclimation but through different mechanisms that in the long term can influence their success and survival. The short duration of the heat exposure does not allow to ascertain the more efficient strategy to cope with longer lasting heat stress, for which longer experiments are needed. However, the higher sensitivity of *P. oceanica* to heat stress, observed in this study, if confirmed as a widespread characteristic of the species, can affect its future distribution. *C. nodosa*, as well as invasive macrophythes coming from warmer environments such as the seaweed *Caulerpa cylindracea*^60^ and the seagrass *Halophila stipulacea*^61^, could benefit from *P. oceanica* regression to colonize new areas causing species substitution and habitat shift. This substitution in species would have dramatic consequences in ecosystem structure and functioning due to their different engineering capacity and ecological functions^62^.

There is an urgent need to determine the ability of Mediterranean seagrasses to acclimate to longer heat exposures through manipulative experiments but also to include in the experiments plants coming from populations under contrasting thermal environments to improve coastal management and to better predict the evolution of Mediterranean coastal ecosystems in the coming decades. Finally other kinds of stressors (e.g. lowered salinities, acidification and light reduction) are also expected to occur in the coming decades, and therefore combined effects of environmental stressors on Mediterranean seagrasses also need to be assessed.

## Methods

### Plant collection and thermal characterization of the water column

On the 15th October 2014, large rhizome fragments of *P. oceanica* and *C.nodosa* were collected by divers from wide and well-preserved meadows, located nearby (<200 m) off the SE coast of Spain (Isla Grosa; 37°43′N, 00°42′W). *P. oceanica* was collected at two contrasting depths (5 m and 25 m) that represent the extremes of the bathymetric distribution of the species in the region, while *C. nodosa* was collected only at 5 m depth. The last species has a shallower distribution in most of its Mediterranean areal, maybe due to its subtropical origin that limits its capacity to colonize deep-cold waters. In order to reduce the likelihood of sampling the same *P. oceanica* genotype twice, plant fragments were collected separated by a distance of at least 65 m, in accordance to previous estimations of the neighbourhood size for the species^63^ and to previous samplings performed on the same population here studied (unpublished data). Plant fragments, consisting of an apical-portion of horizontal rhizomes bearing a large number of vertical shoots, were transported into coolers to the wet lab facilities of the Spanish Institute of Oceanography of Murcia within 2 hours after collection.

Temperature data were provided by the Spanish Oceanography Institute which has a local network of underwater temperature sensors (HOBO Water Temp Pro v2) installed along a bathymetric gradient (i.e. at 5, 12, 20 and 32 m depth) in the study area since 2012. Temperature data are recorded with a frequency of 10 minutes. Data of 2013 and 2014 were used to characterize the thermal structure of the water column and thus the thermal regimen in which collected plants grow.

### Experimental design

*P. oceanica* plant fragments of similar size and bearing a similar number of shoots (i.e. 30–35 and 50–60 shoots for deep and shallow fragments, respectively) were carefully selected. Plant portions were attached by clamps to a rigid plastic mesh and placed in individual and independent 120 L tanks (i.e. 8 tanks for each shallow and deep meadow depth) without adding sediments to prevent possible toxic effects of heat-enhanced sulfide intrusion in the plant tissue^64^. *C. nodosa* fragments were divided into rhizome sections containing 3–5 shoots and randomly planted in plastic trays with washed and dried sediments from the collection site due to the short duration of the experiment and the much higher resistance of the species to sediment anoxia^65^. Trays were placed in eight independent 120 L tanks in a sufficient number to cover all analytical needs. The experimental system of tanks has previously been described^66^. Each tank was provided by independent light source (300 W or 600 W metal halide lamp, Agrolite) and air and submersible pumps for adequate aeration and circulation of seawater to ensure water flow over the leaves and homogeneous temperature within tanks. Irradiance levels in experimental tanks were adjusted according to the origin of plants: 250 ± 20 μmol photons m^−2^ s^−1^ above the canopy of shallow plants and 65 ± 10 μmol photons m^−2^ s^−1^ for deep plants, both with a 8 h:16 h light:dark photoperiod. Water temperature was independently controlled in each tank by a highly precise automated system designed *ad hoc* for the laboratory facilities as previously described elsewhere^67^. The system utilized allowed an accurate control of the water temperature in tanks (±0.2 °C) and temperature was checked twice a day along the course of the experiment by using a handheld mercury thermometer. Salinity within tanks was also checked twice a day along the experiment and maintained within the range 37.3–37.7 ppt by adding purified water to compensate for evaporation. Water quality was maintained in tanks by partially (20–30%) renewing water every 2–3 days.

During a 10d acclimation period water temperature in all tanks was maintained at 24 °C according to natural field values at the moment of sampling (Fig. 1). Subsequently, temperature in half of the tanks containing each species and ecotypes was increased at a rate of 0.5 °C h^−1^ to 32 °C to induce plants to express their mechanisms of response. Selected temperature is within the maximum summer seawater temperature projected for the Mediterranean Sea by the end of the twenty-first century^68^. Plants exposure to heat stress lasted 5-days after which temperature returned again to control levels to determine species/ecotypes capacity for resilience after a recovery period of 5 days. Photo-physiological and transcriptomic plant variables were determined at the end of the acclimation period (T0; only photo-physiological variables), after 24 h (T1) and 5d (T2) of heat exposure and finally at the end of the recovery period (T3). Measures of each plant variable were performed on each experimental tank (n = 4), which is our true experimental replicate, on randomly selected shoots with healthy leaves (i.e. without wounds or herbivore damage) avoiding apical shoots to prevent the influence of apical dependence on plant responses^69^. Gene expression of one randomly picked shoot per tank (n = 4) was assessed at each time point. Photophysiological measures, including respiration, were determined on two shoots per tank and then averaged to be used as individual replicates. Within each shoot, measurements and analyses were always performed on mature leaf tissues of leaf rank 2–3 for *P. oceanica* and rank 1–2 for *C. nodosa*.

### Rates of photosynthesis and respiration

Photosynthetic and respiratory rates were polarographically determined on leaf segments of approximately 2 cm^2^ in area in a DW3 chamber (Hansatech Instruments Ltd, UK) housing a Clark type O_2_ electrode and connected to a controlled temperature circulating bath. Incubations were carried out under the same temperature of the experimental treatments. Dark respiration rates were measured by maintaining the leaf segments in the dark for 10 min. Net oxygen production was then determined at 65 and 250 μmol photons m^−2^ s^−1^ irradiances using a 36 red LED’s light source (LH36/2R, Hansatech Instruments Ltd). Photosynthetic and respiratory rates (μmol O_2_ g^−1^FW h^−1^), and a proxy of the daily leaf metabolic carbon balance (gross-P_max_:R_d_ratio) was calculated for each incubation.

### Chlorophyll a fluorescence parameters

A diving PAM fluorometer (Walz, Germany) was used to characterize the functioning of the photosynthetic apparatus at the PSII level. The basal fluorescence (F_0_) and the maximum photochemical efficiency of PSII (F_v_/F_m_) were measured on night dark-adapted leaves by the saturation pulse method. The rapid light curves method were subsequently performed after 3 hours of illumination to calculate non-photochemical quenching (NPQ) and electron transport rate (ETR) of plants^70^. After each RLC the absorption factor of each leaf was estimated by placing the fluorometer quantum sensor directly behind the leaf and determining the reduction in irradiance and then used to calculate absolute ETR^71^.

### Gene expression analysis (RT-qPCR)

Reverse Transcription – quantitative Polymerase Chain Reaction (RT-qPCR) analysis was used to assess differences in gene expression of target genes following methods described elsewhere^72^. After extraction, total RNA quantity and purity was assessed by a Nanodrop spectrophotometer (NanoDrop Technologies) and the quality evaluated by 1% agarose gel electrophoresis. Subsequently, RNA (500 ng) was reverse-transcribed in complementary DNA (cDNA) with the iScriptTM cDNA Synthesis Kit (Bio-Rad) using the GeneAmp PCR System 9700 (Perkin Elmer).

Genes of interest (GOI) were selected according to the three major stress-sensitive sites in the photosynthetic machinery: The PSII, the electron transport chain and ATP generator and the carbon assimilation processes^30^. Several general stress genes within the heat shock proteins and heat shock transcriptional factors families were also analyzed. Due to the lack of complete genome/transcriptome of both species not all GOI could be designed for both species. Particularly, we selected genes coding for a putative PSII protein D1 (psbA) and D2 (psbD), a putative chloroplastic ATP synthase subunit alpha (atpA), a putative chloroplastic ferredoxin-1 (FD), a putative RuBisCO small subunit (RBCS), a putative RuBisCO large subunit (rbcL), a putative chloroplastic RuBisCO activase (RCA), three putative heat shock proteins (HSP70, HSP90 and one small heat shock protein) and three heat shock transcriptional factors (HSF1, HSF5 and HSF8). As potential reference genes we considered 60S ribosomal protein L23 (L23), 18S ribosomal RNA (18S), ubiquitin C (NTUBC), eukariotic initiation factor 4A (EIF4A) and elongation factor 1-alpha (EF1A).

Most of the primers were already available in the literature or newly designed from transcriptional resources available for both species (RNAseq, unpublished data) (Table 1). Primers were designed using the software Primer3 v. 0.4.0 ( http://frodo.wi.mit.edu/primer3/). Amplification efficiency of primer pairs was in every case >0.92% (R > 0.96) as estimated from the slopes of standard curves of the threshold cycle (CT) of five cDNA dilutions using the equation E = 10^−1/slope^.

RT-qPCR reactions were performed in a Viia7 Real Time PCR System (Applied Biosystem) using Sybr Green as fluorescent detection chemistry^72^. RT-qPCR reactions were conducted in triplicate, and in each assay three no-template negative controls for each primer’s pair were included.

After normalizing by each primer efficiency, the negative differences in CT values between reference genes and each GOI were calculated to obtain −ΔCT for each analyzed sample. Fold expression change were calculated for graphical purposes according to the equation: Fold expression change = ±2^(│(−ΔCTtreatment)−(−ΔCTcontrol)│)^.

L23 and NTUBC were selected as reference genes for *P. oceanica* and EIF4A for *C. nodosa*, according to three different software (BestKeeper, geNorm, and NormFinder) and in agreement with their use as reference genes in previous studies on the same and other seagrass species^20^,^29^,^73^,^74^.

### Statistical analysis

Temperature time series along the bathymetric gradient were represented and analyzed with MatLab. In order to explore the effects of short-term heat stress on seagrass photophysiology, 2-way ANOVA was performed for each sampling time with treatment (2 levels) and species/ecotypes (3 levels) as fixed factors. Similarly, 2-way ANOVA was conducted to assess heat stress effects on the level of expression of selected GOIs except for those GOIs only analyzed in *C. nodosa* for which one-way ANOVA was employed with treatment as the fixed factor. Before carrying out the analyses, data were checked for the assumptions of normality and homoscedasticity and log- or root-transformed when necessary. A post-hoc mean comparison test (Student-Newman-Keuls, SNK) was performed when significant differences were found for the factors (p < 0.05). A principal component analysis (PCA) was performed with T2 gene expression data (−ΔCT values) to explore general patterns in the response to heat among species and ecotypes.

## Additional Information

**How to cite this article**: Marín-Guirao, L. *et al*. Physiological and molecular evidence of differential short-term heat tolerance in Mediterranean seagrasses. *Sci. Rep.* **6**, 28615; doi: 10.1038/srep28615 (2016).

## Supplementary Material

Supplementary Information

## Figures and Tables

**Table 1:** List of reference and genes of interest analyzed in *P.oceanica* (5 and 25 m) and *C. nodosa* (5 m) plants.

Category	Abbrev.	Gene full name	Primers sequence 5′->3′ (F/R)	Accession	*Z. marina* best hit	Score	e-value
Photosystem II	psbA	Photosystem II protein D1	P: GACTGCAATTTTAGAGAGACGC/CAGAAGTTGCAGTCAATAAGGTAG	P: KC954695	ZosmaCg00300	708	0.00
			C: GACTGCAATTTTAGAGAGACGC/CAGAAGTTGCAGTCAATAAGGTAG	C: KT200596	ZosmaCg00300	710	0.00
	psbD	Photosystem II protein D2	P: CCGCTTTTGGTCACAAATCT/CGGATTTCCTGCGAAACGAA	P: KC954696	ZosmaCg00540	706	0.00
			C: CCGCTTTTGGTCACAAATCT/CGGATTTCCTGCGAAACGAA	C: KT200597	ZosmaCg00550	949	0.00
Electron transport chain	ATPa^P^	ATP synthase subunit alpha, chloroplast.	P: TATCCGGCGATCTCTTCAAT/AATTCGCGTAATCGTTGACC	Zoma_Contig753^+^	ZosmaCg00390	971	0.00
	FD*	Ferredoxin, chloroplastic	P: TCAGACTGGGGGTAAGCAAC/TCTACATCCTCGACCACTGC	P: GO348399.1	Zosma196g00110	212	6E-56
			C: ATGGTGAGCACCCCCTTC/GGGTGACGAGCTTGACCTT	C: KT200600	Zosma196g00110	158	5E-40
Carbon assimilation	RCA^P^	RuBisCO activase	CTGTACGCCCCTTTAATTCG/TGACCAGGGAAGGTATCGAC	P:KU994744	Zosma88g00030	744	0.00
	rbcL	RuBisCO large subunit	P: GCTGCCGAATCTTCTACTGG/CACGTTGGTAACGGAACCTT	P: U80719.1	ZosmaCg00710	934	0.00
			C: GCTGCCGAATCTTCTACTGG/CACGTTGGTAACGGAACCTT	C: U80688.1	ZosmaCg00710	538	6E-154
	rbcS*	RuBisCO small subunit	P: AGCATGGTAGCACCCTTCAC/GGGGGAGGTATGAGAAGGTC	P: GO346679.1	Zosma15g00370	330	1.00E-91
			C: TAAGTCGTCCTCCGCCTTC/GGGGGAGGTACGAGAATGTC	C: KT200584	Zosma15g00370	100	1E-22
Heat shock proteins	HSP70^P^	HSP70	P: TCACCAAGTAACTGCCCATA/CCAAGATGTACCAGGGTGC	P: KU994743	Zosma118g00060	1239	0.00
	HSP90*	HSP90	P: CTCCATCTTGCTTCCCTCAG/TCAGTTTGGAGGAACCGAA	P: GO349004.1	Zosma82g00590	1263	0.00
			C: GGACCGCTAACATGGAAAGA/AGGCTGAAGCCAGAGGTGAG	C: KU994740	Zosma82g00590	827	0.00
	SHSP^P^	SH stress protein	P: ACCGGAGGATGTGAAGATTG/AGCTTGCTGGACAAGGTGAT	P: KT159951	Zosma8g01500	101	1E-22
			C: ACCGGAGGATGTGAAGATTG/AGCTTGCTGGACAAGGTGAT				
Heat shock factors	HSFA1^C^	Heat shock factor A1	C: TGAAATGGGAAGCAGGATTG/TTCAAGCTGGCTTGTTAGAT	C: KU994741	Zosma177g00250	55.1	6.0E-09
	HSFA5^P^	Heat shock factor A5	P: GCTCCAACAACTCCAGCTTC/CCCCTTCACAAACTCGTCAT	P: KT159952	Zosma5g02290	312	1E-85
	HSFA8^C^	Heat shock factor A8	C: GGGAGGAGGAAATTGAGAGG/GCAAAATTGGAGAGCAATGC	C:KU994742	Zosma189g00520	53.9	1.0E-08
Reference	18S	18S Ribosomal RNA	P: AACGAGACCTCAGCCTGCTA/AAGATTACCCAAGCCTGTCG	P: AY491942.1			
			C: AACGAGACCTCAGCCTGCTA/AAGATTACCCAAGCCTGTCG	C: KT200607			
	L23^P^	60s ribosomalprotein L23	P: AAAGATACAGGCTGCCAAGG/TGGTCCAACTTGTTCCTTCC	P: GO347779			
	EF1A^P^	Elongation factor 1-alpha	P: GAGAAGGAAGCTGCTGAAATG/GAACAGCACAATCAGCCTGAG	P: GO346663			
	NTUBC^P^	Ubiquitin-conjugating enzyme	P: TCTGCTCGATTCCGAGTTTT/GCTTGAAGTCCCTCATCAGC	P: GO347619			
	eIF4A	Eukariotic initiation factor 4A	P: TTCTGCAAGGGTCTTGACGT/TCACACCCAAGTAGTCACCAAG	P: KU994745			
			C: TTCTGCAAGGGTCTTGACGT/TCACACCCAAGTAGTCACCAAG	C: KT200591			

Category, abbreviation, full name, primers sequences and GenBank accession number are shown. Blast results (including best hits, scores and e-values) of genes of interest blasted against *Z. marina* genome ( http://bioinformatics.psb.ugent.be/orcae/overview/Zosma) are also shown. ^P^ and ^C^: primers only analyzed in *P. oceanica* and *C. nodosa*, respectively. *the same gene was analyzed in both species with specific pair-primers. ^+^from Dr.Zompo database.
