# Supplementary material for: Physiological and molecular evidence of differential short-term heat tolerance in Mediterranean seagrasses
Source: Sci Rep. 2016 Jun 27;6:28615. doi: 10.1038/srep28615 (PMC4921816; doi:10.1038/srep28615)
Supplement: Supplementary Information [file srep28615-s1.doc]

**Physiological and molecular evidence of differential short-term heat tolerance in Mediterranean seagrasses**

Lazaro Marín-Guirao^1*^, Juan M. Ruiz^2^, Emanuela Dattolo^1^, Rocio Garcia-Munoz^2^& Gabriele Procaccini^1^.

^1^Integrative Marine Ecology, Stazione Zoologica Anton Dohrn, Villa Comunale, 80121 Napoli, Italy.

^2^ Seagrass Ecology Group, Oceanographic Center of Murcia, Spanish Institute of Oceanography C/ Varadero, 30740 San Pedro del Pinatar, Murcia, Spain

**Corresponding author:** maringuirao@gmail.com

**Supplementary information**

**Supplementary Figure S1.** Ordination diagram of principal component analysis (PCA) performed with gene expression data (-ΔCT) measured in shallow *C. nodosa* (triangles) and *P. oceanica* (circles) and deep *P. oceanica* (squares) from the control (blue symbols) and heat stress (red symbols) treatments at T2 (5d heat exposure). Only a limited set of markers were included in the analysis since not all GOI could be designed for both species due to the lack of complete genome/transcriptome of both species (as described in material and methods section).

**
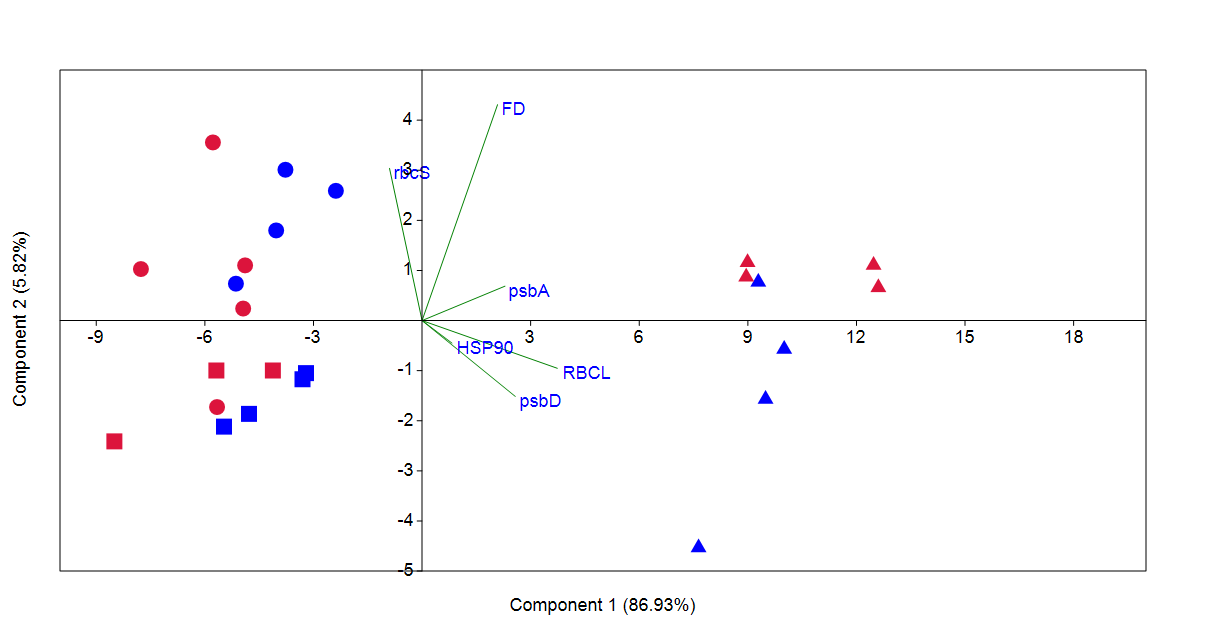
**

**Supplementary Table S1.** Results of the ANOVA analyses for testing for the effects of heat stress on parameters derived from the P-E curves (Photosynthetic and respiratory rates and carbon balance) and from the chlorophyll *a* fluorescence measures (maximal photochemical efficiency of PSII, basal fluorescence, non-photochemical quenching and electron transport rate) along the course of the experiment. T0= end of the acclimation period; T1= 24h of heat exposure; T2= 5d of heat exposure; T3= 5d of recovery.Significant effects are indicated in bold.

**Supplementary Table S2.** Results of the ANOVA analyses for testing for the effects of heat stress on the level of expression of photosynthesis-related genes selected according to the major sensitive sites of the photosynthetic apparatus: Photosystem II (psbA and psbD), electron transport chain (FD and atpA) and carbon fixing processes (rbcL, RBCS and RCA) along the course of the experiment. T1= 24h of heat exposure; T2= 5d of heat exposure; T3= 5d of recovery. Significant effects are indicated in bold.

**Supplementary Table S3.** Results of the ANOVA analyses for testing for the effects of heat stress on the level of expression of general stress genes: Heat shock proteins (HSP70, HSP90 and SHSP) and heat shock factors (HSFA1, HSFA5 and HSFA8) along the course of the experiment. T1= 24h of heat exposure; T2= 5d of heat exposure; T3= 5d of recovery. Significant effects are indicated in bold.

# 
